# Characterization of TTN Novex Splicing Variants across Species and the Role of RBM20 in Novex-Specific Exon Splicing

**DOI:** 10.3390/genes9020086

**Published:** 2018-02-13

**Authors:** Zhilong Chen, Jiangping Song, Liang Chen, Chaoqun Zhu, Hanfang Cai, Mingming Sun, Allysa Stern, Paul Mozdziak, Ying Ge, Warrie J. Means, Wei Guo

**Affiliations:** 1 College of Animal Science and Technology, Northwest A&F University, Yangling 712100, China; zhilongchen@sina.com (Z.C.); hcai@uwyo.edu (H.C.); 2 Animal Science, University of Wyoming, Laramie, WY 82071, USA; czhu1@uwyo.edu (C.Z.); msun2@uwyo.edu (M.S.); means@uwyo.edu (W.J.M.); 3 Department of Cardiac Surgery, State Key Laboratory of Cardiovascular Disease, Fuwai Hospital, National Center for Cardiovascular Diseases, Chinese Academy of Medical Sciences and Peking Union Medical College, Beijing 100730, China; fwsongjiangping@126.com (J.S.); chenliang_2012@126.com (L.C.); 4 Prestage Department of Poultry Science, North Carolina State University, Raleigh, NC 27695, USA; rastern@ncsu.edu (A.S.); pemozdzi@ncsu.edu (P.M.); 5 Department of Cell and Regenerative Biology, Department of Chemistry, Human Proteomics Program, University of Wisconsin, Madison, WI 53705, USA; ying.ge@wisc.edu

**Keywords:** *TTN*, RBM20, Novex isoforms, cardiomyopathy, alternative splicing

## Abstract

*Titin* (*TTN*) is a major disease-causing gene in cardiac muscle. *Titin* (*TTN*) contains 363 exons in human encoding various sizes of TTN protein due to alternative splicing regulated mainly by RNA binding motif 20 (RBM20). Three isoforms of TTN protein are produced by mutually exclusive exons 45 (Novex 1), 46 (Novex 2), and 48 (Novex 3). Alternatively splicing in Novex isoforms across species and whether Novex isoforms are associated with heart disease remains completely unknown. Cross-species exon comparison with the mVISTA online tool revealed that exon 45 is more highly conserved across all species than exons 46 and 48. Importantly, a conserved region between exons 47 and 48 across species was revealed for the first time. Reverse transcript polymerase chain reaction (RT-PCR) and DNA sequencing confirmed a new exon named as 48′ in Novex 3. In addition, with primer pairs for Novex 1, a new truncated form preserving introns 44 and 45 was discovered. We discovered that Novex 2 is not expressed in the pig, mouse, and rat with Novex 2 primer pairs. Unexpectedly, three truncated forms were identified. One *TTN* variant with intron 46 retention is mainly expressed in the human and frog heart, another variant with co-expression of exons 45 and 46 exists predominantly in chicken and frog heart, and a third with retention of introns 45 and 46 is mainly expressed in pig, mouse, rat, and chicken. Using *Rbm20* knockout rat heart, we revealed that RBM20 is not a splicing regulator of Novex variants. Furthermore, the expression levels of Novex variants in human hearts with cardiomyopathies suggested that Novexes 2 and 3 could be associated with dilated cardiomyopathy (DCM) and/or arrhythmogenic right ventricular cardiomyopathy (ARVC). Taken together, our study reveals that splicing diversity of Novex exons across species and Novex variants might play a role in cardiomyopathy.

## 1. Introduction

Alternative splicing is an important RNA post-transcriptional process that produces multiple protein products from a single gene. Alternative splicing enables eukaryotic organisms to meet the ever-changing demands [1,2,3]. Mis-splicing of this process will cause diseases. Studies have shown that abnormal splicing in the heart is associated with heart disease [4,5]. *Titin* (*TTN*) gene is expressed in the sarcomere of the striated muscle containing 363 coding exons that encode the largest protein found in vertebrate animals so far [6]. Titin (TTN) protein plays a critical role in the elastic recoil of the cardiac myocytes and contributes to diastolic function during the ventricular filling phase [7,8,9,10]. It is increasingly recognized as one of the molecular origins of cardiomyopathies and heart failure [11,12]. 

Encoded TTN proteins range from approximately 3.0 to 3.9 MDa as a result of alternative splicing [13,14]. Six major classes of TTN isoforms have been reported including cardiac forms, N2BA, N2B, skeletal muscle form, N2A and both cardiac and skeletal muscle forms: Novexes 1, 2, and 3 with less abundant in skeletal muscle than in the myocardium [6,13,14,15,16]. In these isoforms, the most alternatively used exons are concentrated in the I-band of the sarcomere, especially in the middle immunoglobulin (Ig) region and PEVK (proline (P), glutamate (E), valine (V), and lysine (K)) domain of the TTN protein across the I-band [16]. Alternative splicing of these regions or domains results in two major classes of TTN isoforms, N2B and N2BA including a unique region N2B and two unique regions N2B and N2A, respectively [17,18]. Moreover, a body of evidence in human patients and animal models has shown that changes of the ratios of these two TTN isoforms are associated with cardiomyopathies and heart failure [19,20,21,22,23,24,25,26,27,28,29,30,31,32,33]. Therefore, it is crucial to understand the molecular mechanisms governing TTN isoform regulation. Especially, splicing of Novex isoforms and their role in cardiomyopathies have not been studied. 

**Figure 1:**
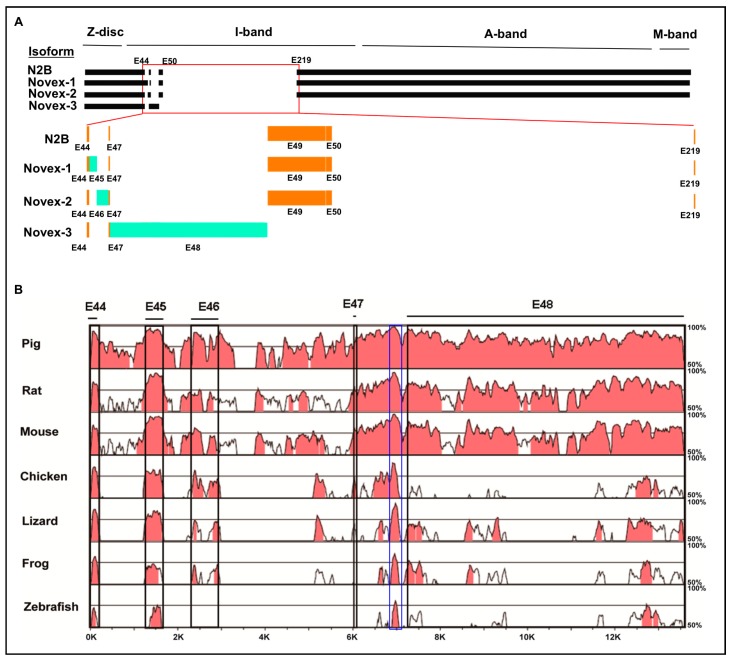
Schematic splicing pattern of *titin* (*TTN)* Novexes 1, 2, and 3 and conserved sequence analysis of other species to human sequence using mVISTA software. (**A**) Schematic splicing pattern of *TTN* Novexes 1, 2, and 3 with comparison to N2B isoform. (**B**) Sequence comparison of exons 44–48 in the Novex-1, -2, and-3 mutually exclusive splicing regions between species. Blue box indicates a highly conserved region between exon 47 and 48. Percentage on the right side represents sequence homology by comparing to human sequence. Large percentage of the red region indicates a high sequence homology to human sequence. E: Exon.

A muscle specific splicing factor RNA binding motif 20 (RBM20) has recently been cloned and identified as a major regulator of *TTN* splicing [19]. RBM20 has been well recognized for its splicing role in N2BA and N2B isoforms, but its splicing role in TTN Novex isoforms remain unknown. Novex variants are produced from mutually exclusive splicing of exons 45, 46, and 48. Novex 1 has exon 45, Novex 2 contains exon 46, and Novex 3 expresses exon 48. Inclusion of Exon 48 in Novex 3 introduces a stop code to generate a truncated isoform with a size of approximately 700 kDa [6]. Except for exons 45 and 46 contained in Novexes 1 and 2, respectively, all other exons are the same as in the N2B isoform (Figure 1A) [16]. Moreover, whether RBM20 regulates these mutually exclusive exons in Novexes 1, 2, and 3 remains completely unknown. Furthermore, whether Novex isoforms are associated with cardiomyopathies has also not been well defined. The objectives of this study were to investigate splicing variants of Novexes 1, 2, and 3 across species and whether RBM20 regulates splicing of Novex-specific exons. Finally, we have analyzed the splicing variants of Novexes 1, 2, and 3 in human heart tissues with cardiomyopathies. 

## 2. Materials and Methods

### 2.1. Experimental Animals and Tissue Samples

Wild type (WT) and homozygous *Rbm20* knockout (KO) rats were employed in the present study. The KO rats were derived from a previously described spontaneous mutant [16,23]. Rats used in the current work were crosses of Sprague-Dawley (SD) X Brown Norway (BN) (All strains were originally obtained from Harlan Sprague-Dawley, Indianapolis, IN, USA). Mice and rats were maintained on standard rodent chow. All animals were maintained in accordance with the Guide for the Care and Use of Laboratory Animals of the National Institutes of Health. All procedures pertaining to animals were approved by the Institutional Animal Use and Care Committee of the University of Wyoming. Pig heart tissues were obtained from the University of Wyoming’s meat science laboratory. Chicken heart tissues were obtained from the North Carolina State University and frog heart tissues were provided by Dan Levy’s lab at the University of Wyoming, WY, USA. Diseased human heart tissues were collected from human transplant heart in the Fuwai hospital in Beijing, China, (ethic approval number 2013-496) and the patient clinical characteristics are in Table S2. The human left ventricular RNA from a de-identified donor heart was obtained from University of Wisconsin-Madison, which was approved by the Institutional Review Board. All tissue samples were snap-frozen in liquid nitrogen and stored at −80 °C.

### 2.2. Genomic Sequences in Multiple Species and Computational Analysis

The *TTN* gene sequences of eight species were obtained through gene database from either University of California Santa Cruz (UCSC, Santa Cruz, CA, USA) [34] or Ensembl [35] genome browsers or National Center for Biotechnology Information (NCBI, Bethesda, MD, USA) nucleotide database [36]. The investigated species were human (*Homo sapiens*, GRCh38), pig (*Sus scrofa*, Sscrofa11), mouse (*Mus musculus*, GRCm38), rat (*Rattus norvegicus*, Rnor_6), chicken (*Gallus gallus*, Gallus_gallus-5), anole lizard (*Anolis carolinensis*, AnoCar2), frog (*Xenopus laevis*, Xenopus_laevis_v2), and zebrafish (*Danio rerio*, GRCz11). The sequences around Novex-1-, -2-, and -3-specific regions were retrieved by basic local alignment search tool (BLAST) [37]. All assembled Novex 1, 2, and 3 sequences were manually edited and checked for consistency. 

Multiple sequence alignments were processed with ClustalX [38] and manually edited to be optimized. Genomic comparisons of cross-species were carried out by an online tool mVISTA [39]. 

### 2.3. RT-PCR and DNA Gel Electrophoresis

Total RNAs from pig, mouse, rat, chicken, and frog heart samples were isolated with Trizol (Invitrogen, Carlsbad, CA, USA) and further treated with RQ1 RNase-free DNase (Promega, Madison, WI, USA) to remove genomic DNA contamination. Reverse transcription (RT) reactions were carried out using ImProm-II Reverse Transcription System (Promega, Madison, WI, USA) with random primers. Standard RT-PCR reactions were carried out using the C1000 Thermal Cycler (BIO-RAD, Hercules, CA, USA). Primer pairs for amplification of Novex-1, -2, and -3 transcripts across different species are listed in Table S1, and the designed primer position is indicated in Figure S1. PCR products were analyzed with the following DNA agarose gel electrophoresis. DNA gel was stained with ethidium bromide and visualized under UV light. The gel images were captured with ChemiDoc Imaging System (Bio-Rad). DNA band density was quantified with NIH ImageJ [40]. 

### 2.4. DNA Sequencing

From the gel electrophoresis, the bands of interest were excised with blade and purified with Wizard SV gel and PCR clean-up system (Promega, Madison, WI, USA). Purified DNA bands were sent out and sequenced to confirm their identity with service provided by Eurofins Genomics. RT-PCR and DNA sequencing of human heart samples with cardiomyopathies were performed in the laboratory of Peking Union Medical College Hospital (Beijing, China).

### 2.5. Statistics

GraphPad prism software (GraphPad Software, Inc., San Diego, CA, USA) was used for statistical analysis. Results were expressed as means ± (Standard Error of the Mean) SEM. Statistical significance between two groups was determined using an unpaired Student’s *t*-test or a Mann–Whitney test. The Kruskal–Wallis test was used to compare means between more than two groups. Significance considered as probability values of *p* < 0.05 are indicated by one asterisk; *p* < 0.001 is indicated by two asterisks; *p* < 0.0001 is indicated by a pound sign in the figures.

## 3. Results

### 3.1. Cross-Species Comparison of TTN Isoform Novex 1, 2, and 3 Splicing Variants

Titin isoforms Novexes 1, 2, and 3 contains three isoform-specific mutually exclusive Exons 45, 46, and 48, respectively (Figure 1A) [41]. These three isoforms could be detected in a healthy human left ventricle by RT-PCR, but it is unknown whether these isoforms are conserved in other species. To test this, we used the human sequence as a reference sequence and performed multiple genomic comparisons of Novex 1, 2, and 3 region across multiple species including pig, rat, mouse, chicken, lizard, frog, and zebrafish by an online tool mVISTA [39] analysis (Figure 1B). The in silico analysis from exons 44 to 48 in Novex-1, -2, and -3 splicing regions indicated that Novex 1 (exon 45) is more highly conserved than Novex 2 (exon 46) and Novex 3 (exon 48) across species (Figure 1B). More importantly, a highly conserved region (highlighted in the blue box) between exons 47 and 48 was discovered across all analyzed species (Figure 1B). This newly discovered conserved region was characterized using RT-PCR and DNA sequencing with Novex-1-, -2-, and -3-specific exons across species and determined the splicing variants across the species in the rest of this study.

### 3.2. Splicing Variants of the Novex-1-Specific Exon 45 across Species

**Figure 2:**
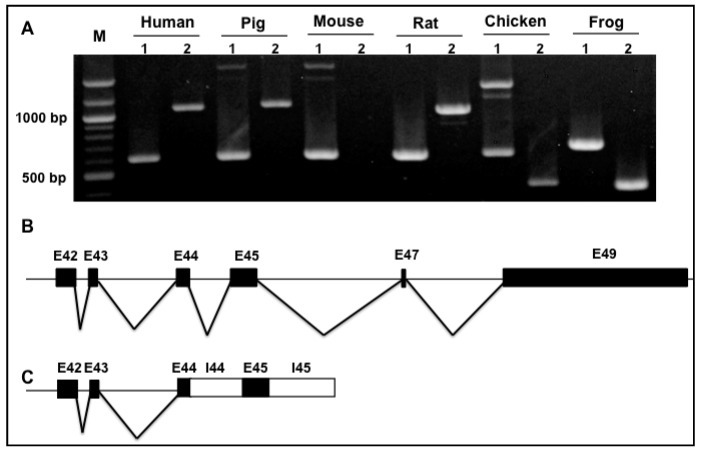
Splicing analysis of *TTN* Novex 1 between species. (**A**) PCR products with primers spanning exons 42–45 (1) and Exons 45–49 (2); expression between exons 42–45 is consistent across species, while different expression pattern is observed between exons 45 and 49. (**B**) Schematic diagram of the reported Novex 1 splicing pattern in all species but mouse. (**C**) A newly identified truncated splicing form with Intron 44 retention in pig, mouse, and chicken heart. M: DNA marker; E: Exon; black boxes: Exons; white boxes: Intron retention; black lines: Introns.

Primer pairs spanning exons 42–45 (1) and exons 45–49 (2) were designed to examine splicing variants of exons from 42 to 49 across species. RT-PCR with RNAs from the left ventricular tissues of pig, mouse, rat, chicken, frog, and healthy human heart indicated that a PCR product consistent with reported Novex 1 isoform was observed with primer pair spanning exon 42 to 45 (1) across species, but an additional larger band was observed in pig, chicken, and mouse heart (Figure 2A). DNA sequencing showed that this is a truncated isoform with intron 44 retention, which is a newly identified isoform. A schematic diagram of this new variant is indicated in Figure 2C. With primer amplifying Novex 1 sequence from Exons 45 to 49 (2), human, pig, and rat hearts express the alternatively used exons 45–47–49 (Figure 2A,B). No PCR products were detected in mouse heart, and the smaller PCR products amplified in chicken and frog heart were observed (Figure 2A and Table S1). However, the smaller products are caused by species sequencing difference, not splicing pattern changes.

### 3.3. Splicing Variants of the Novex-2-Specific Exon 46 across Species

**Figure 3:**
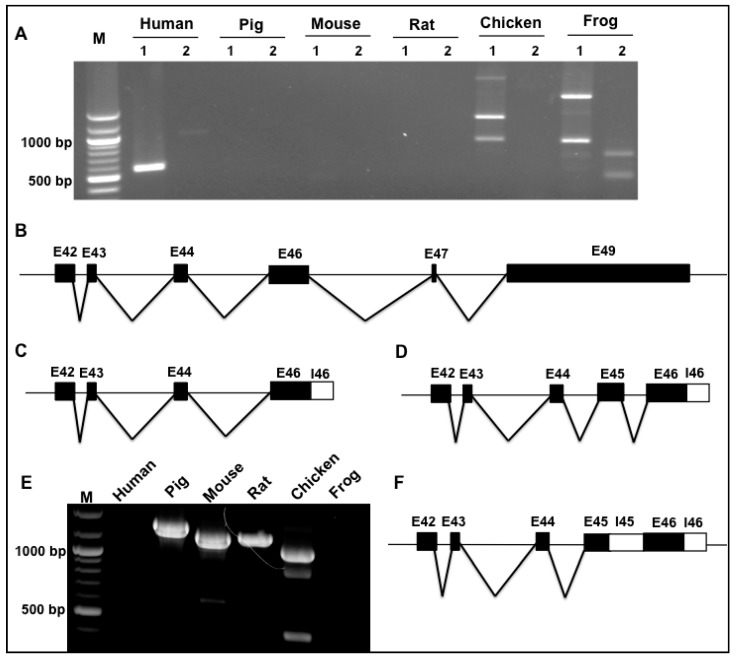
Splicing analysis of *TTN* Novex 2 between species. (**A**) PCR products with primer pairs spanning exon 42 and 46 (1) and exon 46 and 49 (2); human, chicken, and frog express Exons 42–46 and Exons 46–49 with a lower level; no products could be detected in pig, mouse, or rat. (**B**) Schematic diagram of the reported Novex 2 splicing pattern in human, chicken, and frog heart. (**C**) Schematic diagram of PCR results; a truncated form with Intron 46 retention was found in human and frog heart. (**D**) Schematic diagram of PCR results; a new truncated form with co-expression of exons 45 and 46 and Intron 46 retention was observed in chicken and frog hearts. (**E**) PCR products with primer spanning exon 45 and Intron 46 between species; DNA band was observed in pig, mouse, and rat heart. (**F**) Schematic diagram of a newly identified truncated form with co-expression of Exons 45 and 46 and Introns 45 and 46 retention in pig, mouse, rat, and chicken. M: DNA marker; E: Exon; black boxes: Exons; white boxes: Intron retention; black lines: Introns.

We next investigated the Novex 2 splicing pattern across species. We designed primers spanning exons 42–46 (1), and exons 46–49 (2) to determine splicing variants of Novex 2 exons from 42 to 49 across species. Intriguingly, previously reported Novex 2 contains exons 42–43–44–46–47–49, which is a major splicing form (Figure 3A,B), but we found that this splicing pattern of Novex 2 was only expressed in human and frog hearts with low expression level, and no PCR products can be detected in pig, mouse, and rat hearts (Figure 3A). It appears that the splicing pattern of Novex 2 mainly exhibits shorter transcript variants that introduce the stop code after the Novex-2-specific exon 46 in human, chicken, and frog hearts (Figure 3C). Unexpectedly, a new splicing pattern was revealed to exhibit co-expression of Novex-1-specific exon 45 and Novex-2-specific exon 46, introducing a stop code in chicken and frog hearts (Figure 3D). 

To further test whether co-expression of Novex-1-specific exon 45 and Novex-2-specific exon 46 occurs across species, primer spanning exon 45 and intron 46 were designed. RT-PCR results indicated that PCR products were detected in pig, mouse, rat, and chicken hearts, but not in human or frog hearts (Figure 3E). DNA sequencing with these bands showed that, except for co-expression of exons 45 and 46, intron 45, and part of intron 46 were reserved (Figure 3F). Even though reported Novex 2 in Figure 3B was not detected in pig, mouse, and rat, but co-expression of Novex-1-specific exon 45 and Novex-2-specific exon 46 with retention of introns 45 and 46 was significantly expressed in these species and may produce a truncated form. 

### 3.4. Splicing Variants of the Novex-3-Specific Exon 48 across Species

**Figure 4:**
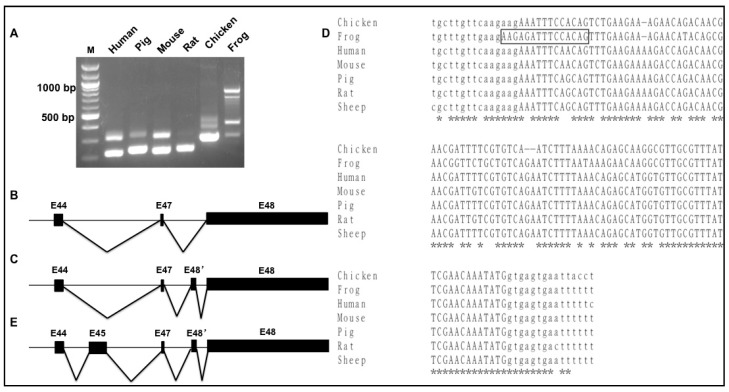
Species difference in splicing of *TTN* Novex 3 between species. (**A**) PCR products with primer pair spanning exons 44 and 48; the lower band was expressed across all species with a lower level in frog. An additional band was amplified in all species with a lower level in rat; in frog heart, an even larger product appears. (**B**) Schematic diagram of splicing pattern of the reported Novex 3. (**C**) Schematic diagram of a newly identified isoform of Novex 3 with an additional exon 48′ insertion in all species with a lower expression in rats between exon 47 and 48. (**D**) Sequencing of exon 48′ and alignment of new Novex 3 isoform between species; the size of exon 48′ is either 85 or 100 bp due to alternatively used 15 bp sequence highlighted in black box, and this sequence is conserved between species. (**E**) Schematic diagram of another new splicing isoform of Novex 3 with co-expression of exons 45 and 48 and additional exon 48′ insertion. E: Exon, M: DNA marker.

To determine whether the Novex 3 undergoes diverse splicing across species, we employed primer pair spanning exons 44 and 48 to amplify the splicing region covering specific Novex exons 45, 46, and 48. Across species, it was discovered that all chosen species express a common Novex 3 variant connecting Exons 44–47–48 that encodes a truncated protein as reported previously [6] (Figure 4A,B). Interestingly, a new variant harboring a new exon named 48′ between exons 47 and 48 was present across all chosen species (Figure 4A,C). DNA sequencing indicated that the size of this additional exon 48′ is either 85 or 100 bp in length, with a 15 bp sequence alternatively used as exon or intron (highlighted in black box) (Figure 4D). The additional exon harbors a high sequence similarity among the species as predicted in Figure 1B, highlighted in the blue box. Inclusion of this new exon 48′ leads to a frame-shift mutation of Novex 3 that encodes a shorter protein than the reported Novex 3 isoform (≈700 KDa) [6]. Furthermore, we identified that, in the frog, an additional Novex 3 splicing variant exists with co-expression of the Novex-1-specific exon 45, the additional exon 48′ and the Novex-3-specific exon 48 (Figure 4E). This variant also encodes a truncated protein shorter than the reported Novex 3 isoform (≈700 KDa).

### 3.5. Splicing Regulation of RBM20 in the Novex 1, 2, and 3 Isoforms 

**Figure 5:**
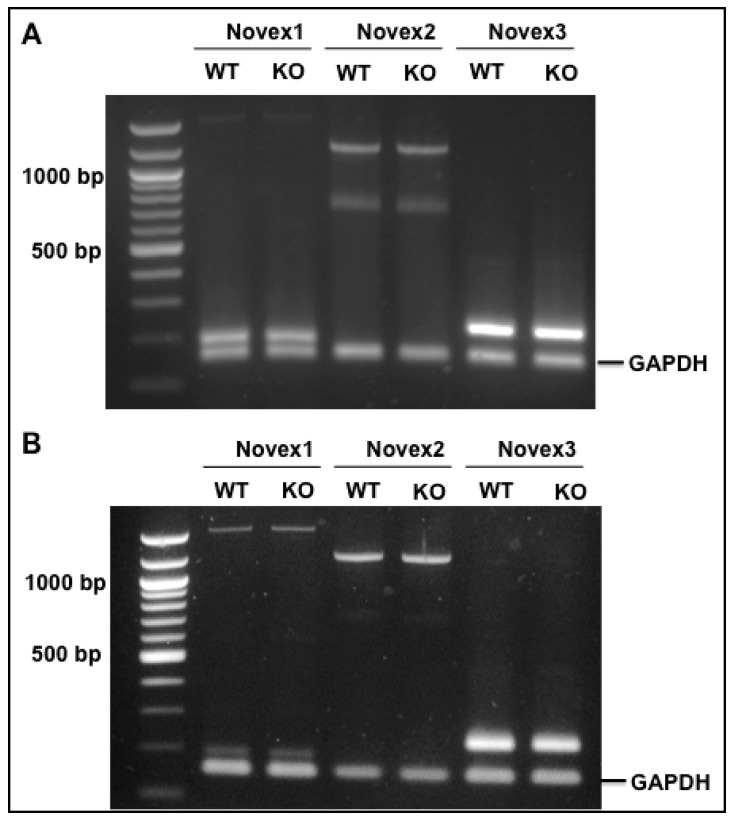
RBM20 regulation of *TTN* Novex alternatively used exons. (**A**) PCR products with primers spanning exons 44–45 (Novex 1), Exons 44–46 (Novex 2), and exons 44–48 (Novex 3) in rat fetal heart of WT and KO; no splicing difference was found between WT and KO. (**B**) PCR products with the same primers in adult rat heart of WT and KO; no difference between WT and KO was found. GAPDH: PCR internal control. M: 1 Kb DNA marker; WT: Wild type; KO: Knockout.

RBM20 is a muscle-specific splicing factor with the highest expression in heart muscle that mainly regulates *TTN* splicing [19]. *Titin* has five major isoforms N2B, N2BA, Novex 1, Novex 2, and Novex 3 as a result of alternative splicing in the heart. RBM20 has been found to mainly regulate the middle Ig region and PEVK region in these five isoforms [19,42], but it has not been determined to regulate splicing occurring in the N-terminal of the I-band region, which is the specific splicing region for Novexes 1, 2, and 3 [6]. To test whether RBM20 regulates splicing of exons 45 (Novex 1), 46 (Novex 2), and 48 (Novex 3), we used primers spanning exons 44–45 for Novex 1, exons 44–46 for Novex 2, and exons 44–48 for Novex 3 to detect splicing difference with fetal and adult heart tissues between WT and *Rbm20* KO rats, because the splicing pattern differs between fetal and adult heart [13,14,43]. *Glyceraldehyde-3-Phosphate dehydrogenase* (*GAPDH*) was used as an internal control. According to the Novex 1, 2, and 3 splicing pattern identified above, we expected that primers 44–45 would amplify two products, the smaller one with exons 44 and 45 and the larger one with intron 44 inserted between exons 44 and 45, that primers 44–46 would amplify a larger product with exon 45 insertion between exons 44 and 46 because alternative splicing of exons 44–46 does not occur in rats, and that primers 44–48 would show one band with a direct connection of exons 44-47-48 on agarose gel since the additional exon 48′ is expressed at a very low level in rats. The results supported the predicted sequences (Figure 5A,B), and no difference was found between WT and KO rat fetal and adult hearts, suggesting that RBM20 is not a regulator of splicing in the specific alternatively used exon regions of Novex 1, 2, and 3.

### 3.6. Splicing Pattern of the Novexes 1, 2, and 3 in Human Cardiomyopathies 

**Figure 6:**
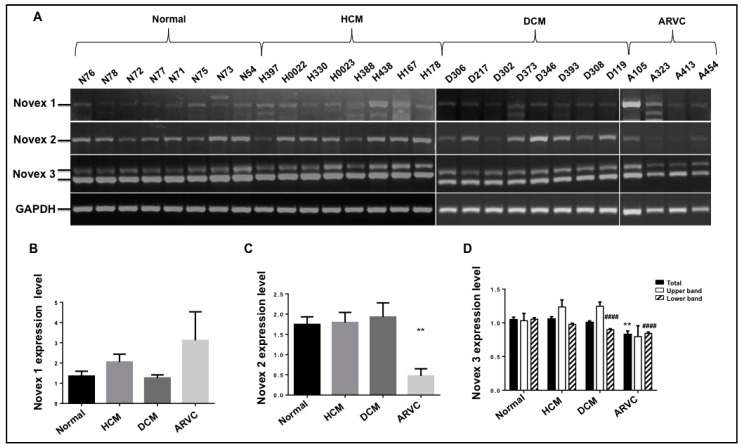
Splicing pattern and expression level of Novex isoforms in human cardiomyopathies. (**A**) PCR products with primers spanning exons 44–45 (Novex 1), exons 44–46 (Novex 2), and exons 44–48 (Novex 3) in normal and diseased hearts; no splicing pattern difference was found between normal and diseased hearts, but expression levels of Novex isoforms was observed. (**B**) Quantification of DNA band intensity of Novex 1 in HCM, DCM, and ARVC. (**C**) Quantification of DNA band intensity of Novex 2 in HCM, DCM, and ARVC. (**D**) Quantification of DNA band intensity of Novex 3 in HCM, DCM, and ARVC; Gapdh: internal control; HCM: hypertrophy cardiomyopathy; DCM: Dilated cardiomyopathy; ARVC: Arrhythmogenic right ventricular cardiomyopathy. Mean ± (Standard Error of the Mean) SEM, ** *p* < 0.001; ^####^ *p* < 0.00001.

In addition, the splicing role of Novexes 1, 2, and 3 has not been well studied. In order to test whether these isoforms are associated with cardiomyopathies, we employed the primers spanning exons 44–45, exons 44–46, and exons 44–48 to assess the splicing pattern and expression level of Novexes 1, 2, and 3 in healthy human heart tissues and heart tissues from hypertrophic cardiomyopathy (HCM), dilated cardiomyopathy (DCM), and arrhythmogenic right ventricular cardiomyopathy (ARVC). The current results demonstrate that Novex 1 produces a major transcript with a reported splicing pattern of exons 44–45–47–49 across all samples, and no expression difference was observed in HCM, DCM, and ARVC when compared to the healthy heart (Figure 6A,B). Novex 2 exhibits a major band in the human heart across all tissues which could be the full length of Novex 2 with exons 44–46–47–49 or truncated Novex 2 or both because primers 44–46 covers both splicing variants. It was discovered that no change in expression was found in HCM and DCM compared to the normal heart, but it is significantly lower in the ARVC heart when compared to the normal heart (Figure 6A,C). Our findings revealed that Novex 3 expresses two products with or without extra exon 48′ insertion (Figure 4), so we expected two PCR bands from agarose gel electrophoresis. With primer spanning exons 44–48, two expected bands were observed in diseased and donor hearts. Quantification of DNA bands with NIH Image J indicated that upper band with Exon 48′ insertion did not differ in all cardiomyopathy samples, but tended to increase in HCM and DCM. The lower band decreased in DCM and ARVC when compared to the normal heart, and total Novex 3 isoform was reduced in hearts with ARVC (Figure 6A,D). These results suggest that Novex isoform changes are mainly associated with DCM and/or ARVC.

### 3.7. In Silico Analysis of Titin Novex-1, -2, and -3 Exons 42–50

The gene structure of human *TTN* gene transcript variants, Novexes 1, 2, and 3 is available in the NCBI database; however, the orthologs of Novexes 1, 2, and 3 in other species have not been reported in previous studies. The exon/intron structure of exons 42–50 presented in Novexes 1, 2, and 3 across species were analyzed using a comparative genomics approach. Specifically, the current study identified the corresponding exon/intron junctions of Novex-1, -2, and -3 variants in pig, rat, mouse, chicken, lizard, frog, and zebrafish (Table 1). Alternatively used exons 45, 46, and 48 were less conserved across all analyzed species, determined by comparison with the constitutive exons around them, and, based on database comparison analysis, Novex-2-specific exon 46 does not express in rat, mouse, or zebrafish (Table 1). However, the current results indicated that Exon 46 does express in mouse and rat, but with co-expression with exon 45 and Intron 45 (Figure 3E,F). It is of interest to note that the Novex-2-specific exon 46 is 576 bp in the human gene database, and we found that it was 573 bp using RT-PCR followed by DNA sequencing (Table 1). However, missing three base pairs does not shift the DNA coding frame. Whether this causes functional changes it needs to be further studied.

## 4. Discussion

The giant multi-functional protein TTN spans half of a sarcomere and connects the Z-band to the M-band that is located in the center of the sarcomere. Two TTN molecules cross-link its C-terminal in the M-band and their N-terminal attaches another TTN’s N-terminal from adjacent sarcomere in the Z-band and thus forms a continuous system along the myofibril [6,44,45]. This structural arrangement enables TTN to function as a molecular blueprint for the maintenance of sarcomere integrity and precise assembly of the regulatory, contractile, and structural proteins located in the sarcomere [16,46,47,48]. However, TTN also plays a role in myocardial passive stiffness due to its elasticity. TTN stiffness is mainly determined by the sizes of its spring-like domain, which changes in sizes resulting from alternative splicing [15,16,47,49,50]. As a result of alternative splicing, TTN produces five major isoforms in the heart including N2B, N2BA, and Novexes 1, 2, and 3. Novexes 1 and 2 are composed of very similar exon numbers to N2B (Figure 1A) except for exons 45 and 46 which are specific exons in Novex 1 and Novex 2, respectively [6,41]. Novex 3 has exon 48 inclusion that introduces a stop codon resulting in an unusually small isoform (≈700 kDa) [6]. These three isoforms have not been well studied in all aspects including their splicing pattern across species, regulatory mechanisms of their specific exon usage, and their functional role across the life course and in heart disease. In our comprehensive analysis of their splicing pattern across species, we revealed a new truncated transcript with Novex-1-specific exon 45, and that Novex-2-specific exon 46 can co-express with Novex-1-specific exon 45. The new findings indicate that retention of Introns 44, 45 and 46 occurs in all truncated new isoforms because the intron retention will cause a reading frame shift and introduce a stop codon. One interesting finding is that an extra exon named 48′ is inserted between exons 47 and 48 in the Novex 3 variant, which causes a reading frame shift and introduces a stop codon that results in even smaller Novex 3 isoform. This new Novex 3 isoform is expressed in almost all species at high levels of expression. 

One goal of the study was to examine a mutually exclusive usage of *TTN* Novex-specific exons. Since the major *TTN* splicing factor RBM20 has been identified, we assume that RBM20 could be the regulator for mutually exclusive splicing of the three Novex variant-specific exons. Using the *Rbm20* KO rat model, it was expected that these three exons should all be included in the spliced mRNA because previous studies have shown that RBM20 inhibits alternative splicing [19,42]. Unexpectedly, it was discovered that RBM20 does not regulate mutually exclusive splicing of the three Novex-variant-specific exons. Our result suggests that some other factors may regulate mutually exclusive exons independently or cooperatively with RBM20, which need to be further studied.

Another major goal of the study was to understand the splicing role of Novex variants in heart function by testing the splicing pattern and expression level with transplant heart tissues of HCM, DCM, and ARVC. It was discovered that the splicing pattern of these three variants has no difference between the healthy and cardiomyopathic heart, and the same variants are expressed in both healthy and disease hearts. However, there appears to be differential expression of the variants in the diseased state. Novex 1 seems unrelated to any cardiomyopathies, and Novex 2 is significant lower in ARVC. Novex 3 is associated with both DCM and ARVC. Particularly, the expression level of Novex 3 without insertion of Exon 48′ is important for cardiomyopathies. 

In summary, this study provides the first evidence that *TTN* Novex variants not only have conventional transcripts as reported previously, but also truncated transcripts with either intron retention or additional exon insertion. The functional significance of these variants is completely unknown, but truncation variants like Novex 3 are short and cannot reach the A-band, so they may adjust the TTN filament system to both three- and two-fold symmetries of thick and thin filaments when co-expressing with the full length TTN [6]. Importantly, *TTN* Novex splicing is not regulated by RBM20, a major *TTN* splicing factor. Although expression levels of Novex variants were changed in cardiomyopathies such as DCM and ARVC, the conclusion that onset and development of cardiomyopathies are associated with Novex isoforms due to sample size limitation cannot yet be drawn. However, the current study provides preliminary information regarding *TTN* Novex splicing and their potential function in cardiomyopathies.

## Figures and Tables

**Table 1:** Sequence comparison of exons 42–50 in Novex and N2B isoforms between species according to the National Center for Biotechnology Information (NCBI) gene database and our PCR product sequencing.

	Regions	Human	Pig	Rat	Mouse	Chicken	Lizard	Frog	Zebrafish
Species									
**Exon42**		**285**	**285**	**285**	**285**	**285**	**285**	**285**	**285**
Intron42		224	222	177	188	100	124	108	83
**Exon43**		**126**	**126**	**126**	**126**	**126**	**126**	**126**	**126**
Intron43		5004	3844	4470	4856	1795	3190	584	634
**Exon44**		**189**	**189**	**189**	**189**	**189**	**189**	**189**	**189**
Intron44		1067	1095	1382	1107	723	1564	559	2350
**Exon45 (Novex 1 exon)**		**375**	**375**	**375**	**375**	**375**	**372**	**375**	**390**
Intron45		744	3990	3779	3997	628	671	571	357
**Exon46 (Novex 2 exon)**		**573 ^a^**				**582**	**558**	**552**	
Intron46		3044				1898	3257	2337	
**Exon47**		**57**	**45**	**57**	**57**	**57**	**57**	**48**	**51**
Intron47		1084	1091	1094	1087	1102	1105	1021	1022
**Exon48 ^b^ (Novex 3 exon)**		**6455**	**6443**	**6266**	**6287**	**6077**	**8039**	**7088**	**6866**
Intron47 ^c^		11,202	12,096	10,890	11,597	11,301	14,386	12,571	8954
**Exon49 (N2B exon)**		**2781**	**2742**	**2628**	**2646**	**2544**	**3846**	**3630**	**3771**
Intron49		780	773	792	775	562	876	1298	919
**Exon50**		**279**	**279**	**279**	**279**	**279**	**279**	**276**	**279**

^a^ Human Novex 2 exon expresses 573 bp instead of 576 bp indicated in the NCBI database. ^b^ Specific Novex 3 exon 48 introduces a stop code. ^c^ Intron 47 is split into two introns by exon 48, but one intron remains in Novexes 1 and 2.

## Supplementary Materials

The following are available online at http://www.mdpi.com/2073-4425/9/2/86/s1, Table S1: Primer information for RT-PCR, Table S2: Human patient clinical characterizations; Figure S1: Primer position in Novex variants.
