# Supplementary material for: Characterization of TTN Novex Splicing Variants across Species and the Role of RBM20 in Novex-Specific Exon Splicing
Source: Genes (Basel). 2018 Feb 13;9(2):86. doi: 10.3390/genes9020086 (PMC5852582; doi:10.3390/genes9020086)
Supplement: Supplementary file 1 [file genes-09-00086-s001.pdf]

Primers for identification of TTN novex 1		
Primer pair	Primer sequence	PCR products sizes (bp)
Human P1	E42-F: CCTGGTACAAGGAAGAGCAG<br>E45-R: GCTTGACAGAGGCTGGATGA	608
Human P2	E45-F: AGCCTCTGTCAAGCCTCAG<br>E49-R: GTTCCAGAGCCATTAGGGTT	1003
Pig P1	E42-F: CCTGGTACAAGGAAGAGCAG<br>E45-R: CAGAGGCTGGATGAAGATGGG	602
Pig P2	E45-F: AAAGCCCATCTTCATCCAGC<br>E49-R: CTCTGCTTTACAGACATAGAGAC	1066
Mouse P1	E42-F: CCTGGTACAAGGAGGAGCAG<br>E45-R: CAGAGGCTGGATGAAGATGGG	602
Mouse P2	E45-F: AAAGCCCATCTTCATCCAGC<br>E49-R: GACTTATTCTTAGGTGAGCGC	767
Rat P1	E42-F: CCTGGTACAAGGAAGAGCAG<br>E45-R: CAGAGGCTGGATGAAGATGGG	602
Rat P2	E45-F: AAAGCCCATCTTCATCCAGC<br>E49-R: CCAGAGCCGTCAGGATTG	1005
Chicken P1	E42-F: TGGTAAACCTGCCCGCTTCT<br>E45-R: TAGGCTGGATAAAGATGGGC	656
Chicken P2	E45-F: CATGCCTAAACCAGAAATCCA<br>E49-R: TTTCTGCTCGCTTTGACTGG	406
Frog P1	E42-F: CCTGCCCGATTTACTACTGT<br>E45-R: CTTGAAATAGGCTGAGTGAAGAC	656
Frog P2	E45-F: TTCACTCAGCCTATTTCAAGC	389

	E49-R: TTGCTTCGGTAGGATTCTGT	
**Primers for identification of TTN novex 2**		
Human P1	E42-F: CCTGGTACAAGGAAGAGCAG<br>E46-R: CTGCCACTGCCTGGGACTT	561
Human P2	E46-F: ACTGTAAAGTGCGGTGACAC<br>E49-R: GTTCCAGAGCCATTAGGGTT	974
Pig P1	E42-F: CCTGGTACAAGGAAGAGCAG<br>E46-R: CTGCCACTGCCTGGGACTT	561
Pig P2	E46-F: CAGGACCTCTCTGTAAAGTGC<br>E49-R: CTCTGCTTTACAGACATAGAGAC	1038
Mouse P1	E42-F: CCTGGTACAAGGAGGAGCAG<br>E46-R: AGGACAGGGTGCAGCATG	717
Mouse P2	E46-F: CTGCACCCTGTCCTGAGTTA<br>E49-R: GACTTATTCTTAGGTGAGCGC	815
Rat P1	E42-F: CCTGGTACAAGGAAGAGCAG<br>E46-R: AGGACAGGGCACGGCATCG	705
Rat P2	E46-F: CCGTGCCCTGTCCTGAGTGA<br>E49-R: CCAGAGCCGTCAGGATTG	1039
Chicken P1	E42-F: TGGTAAACCTGCCCGCTTCT<br>E46-R: TGTAGCTTTCATGGTCAGCC	754
Chicken P2	E46-F: CCAGACATCTCAGAGCACC<br>E49-R: TTTCTGCTCGCTTTGACTGG	532
Frog P1	E42-F: CCTGCCCGATTTACTACTGT<br>E46-R: TGTCTTCATGGCTGAGGC	729
Frog P2	E46-F: CCTCAGCCATGAAGACAGT<br>E49-R: TTGCTTCGGTAGGATTCTGT	491

Human P3	E45-F: CCTGGTACAAGGAAGAGCAG<br>E46-R: CTGCCACTGCCTGGGACTT	1086
Pig P3	E45-F: AAAGCCCATCTTCATCCAGC<br>E46-R: CTGCCACTGCCTGGGACTT	1302
Mouse P3	E45-F: AAAGCCCATCTTCATCCAGC<br>E46-R: AGGACAGGGTGCAGCATG	1187
Rat P3	E45-F: AAAGCCCATCTTCATCCAGC<br>E46-R: AGGACAGGGCACGGCATCG	1185
Chicken P3	E45-F: CATGCCTAAACCAGAAATCCA<br>E46-R: TGTAGCTTTCATGGTCAGCC	1043
Frog P3	E45-F: TTCACTCAGCCTATTTCAAGC<br>E46-R: TGTCTTCATGGCTGAGGC	1039
**Primers for identification of TTN novex 3**		
Primer pair	Primer sequence	PCR products sizes (bp)
Human	E44-F: AGAAGATGAAGGAACTTACACG<br>E48-R: GGATTTGTGGTCTATGTCTTCAG	223
Pig	E44-F: AGCTTTTCCAGAAGATGAAGG<br>E48-R: GCATATCTTTTAGCAGCCATG	240
Mouse	E44-F: AGGACGAGGGAACGTATGC<br>E48-R: CCATCAGGGAGTGGTGGTCTAT	225
Rat	E44-F: CTGAGGACGAAGGAACTTACGC<br>E48-R: CCATCAGGGAGTGGTGGTCTAT	228
Chicken	E44-F: ACTCAGTTTGAAGACACTTACCA<br>E48-R: CTCCAAGACGGACCTACAC	297
Frog	E44-F: GCTGGAAATTGCTGAGGC	300

	E48-R: GACCAGGAACCTATGGAAGA	

**Primers for RT-PCR of Ttn novex in WT and KO rat**		
Primer pair	Primer sequence	PCR products sizes (bp)
Gapdh	Gapdh-F: AACATCATCCCTGCATCCAC<br>Gapdh-R: CATACTTGGCAGGTTTCTCC	154
novex 1	E44-F: CTGAGGACGAAGGAACTTACGC<br>E45-R: CAGAGGCTGGATGAAGATGGG	191
novex 2	E44-F: CTGAGGACGAAGGAACTTACGC<br>E46-R: AGGACAGGGCACGGCATCG	1351
novex 3	E44-F: CTGAGGACGAAGGAACTTACGC<br>E48-R: CCATCAGGGAGTGGTGGTCTAT	228

**Primers for RT-PCR of TTN novex in human cardiomyopathies**		
Primer pair	Primer sequence	PCR products sizes (bp)
GAPDH	GAPDH-F: ACCCACTCCTCCACCTTTGA<br>GAPDH-R: CTCTTCCTCTTGTGCTCTTGC	189
novex 1	E44-F: AGAAGATGAAGGAACTTACACG<br>E45-R: GCTTGACAGAGGCTGGATGA	186
novex 2	E44-F: AGAAGATGAAGGAACTTACACG<br>E46-R: CTGCCACTGCCTGGGACTT	149
novex 3	E44-F: AGAAGATGAAGGAACTTACACG	223/321

	E48-R: GGATTTGTGGTCTATGTCTTCAG	

Table S1. Primer information for RT-PCR

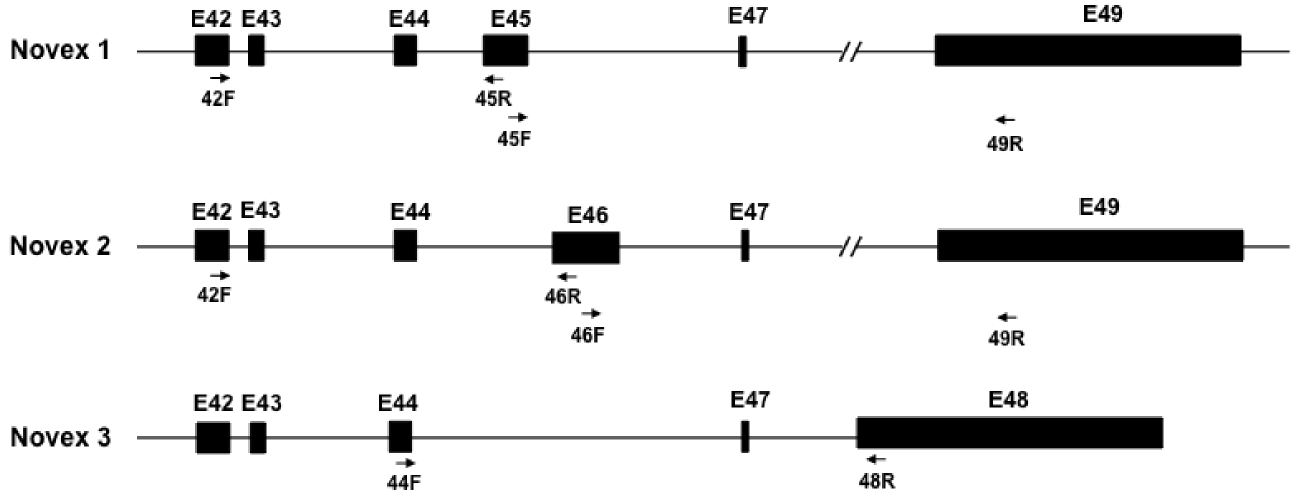

Figure S1. Primer position spanning novex exons.

ID	Diagnosis	gender	age	height	weight	BMI	systolic pressure	diastolic pressure	NYHA	LVEDD (mm)	LVEF (%)	NTproBNP (pg/L)	hsCRP (mg/L)
N76	Brain death	male	37	170	60	20.8							
N78	Brain death	male	35	175	86	28.1							
N72	Brain death	male	28	165	57	20.9							
N77	Brain death	female	38	173	61	20.4							
N71	Brain death	male	32	175	59	19.3							
N75	Brain death	male	25	173	65.0	21.7							
N73	Brain death	female	36	172	75.0	25.4							
N54	Brain death	male	33	164	55.0	20.4							
H397	HCM	female	44	160	56.5	22.1	80	50	IV	7.4	14.6	2779.9	4.18
H0022	HCM	male	36	173	69.5	23.2	90	60	III	6.2	23	4307.3	12.64
H330	HCM	female	54	160	50.5	19.7	110	70	III	5.5	30	1794	7.75
H0023	HCM	male	16	164	56.0	20.8	95	60	III	6.6	23.0	3570.7	
H388	HCM	male	40	169	54.0	18.9	90	60	IV	8.4	22.4	3062.4	0.02
H438	HCM	male	19	174	45.5	15.0	97	65	III	6.8	18.2	3282	4.84
H167	HCM	male	56	177	69.5	22.2	100	60	III	5.5	35.0	2733.5	1.92
H178	HCM	male	60	164	50.5	18.8	125	82	III	6.9	25.2	4960.9	0.01
D306	DCM	male	30	175	58	18.9	94	62	IV	7.2	15	1363.8	0.16
D217	DCM	female	61	162	60	22.9	100	65	III	7.2	20	7298.5	1.96
D302	DCM	female	49	162	54.5	20.8	95	61	IV	6.7	34.0	1429.9	1.39

D373	DCM	male	40	177	63	20.1	91	68	IV	7.4	26	5729.6	13.26
D346	DCM	male	48	164	49.5	18.4	96	65	III	6.2	31	2276.9	3.22
D393	DCM	male	15	173	54	18.0	103	76	IV	7.6	24	2074.3	0.41
D308	DCM	male	41	175	59	19.3	98	58	III	8.2	28	2942.7	7.06
D119	DCM	male	72	172	65	22.0	119	89	III	5.9	37	4016.3	0.68
A105	ARVC	male	43	164	55.0	20.4	117	70	II	5.0	40.0	1996.7	4.26
A323	ARVC	male	36	175	89.0	29.1	95	64	IV	6.1	20.0	4563.9	7.77
A413	ARVC	male	22	170	82	28.4	95	57	IV	4.7	60	1106.3	12.69
A454	ARVC	male	44	175	61.7	20.1	98	61	II	6.3	23.6	6179	12.69

Table S2, clinical characteristics
